# Janus Kinase (JAK) Inhibitors in Rheumatoid Arthritis

**DOI:** 10.7759/cureus.102634

**Published:** 2026-01-30

**Authors:** Khulood K Burashed, Faten A AlAbbasi

**Affiliations:** 1 Medicine, Royal College of Surgeons in Ireland, Busaiteen, BHR; 2 Family Medicine, Hamad Kanoo Health Center, Riffa, BHR

**Keywords:** filgotinib, jak inhibitor, peficitinib, rheumatoid arthritis, tofacitinib, upadacitinib

## Abstract

Rheumatoid arthritis (RA) is a progressive autoimmune disease with systemic involvement and is characterized by synovial inflammation, unremitting joint destruction, and systemic involvement. While biologic disease-modifying antirheumatic drugs (bDMARDs) and conventional synthetic DMARDs (csDMARDs) form the foundation of treatment, limitations such as incomplete efficacy, parenteral administration, and adverse events highlight the need for alternative strategies. Janus kinase inhibitors (JAKis), a newer class of targeted synthetic DMARDs (tsDMARDs), offer the advantages of oral administration, rapid onset, and broad cytokine modulation, thereby potentially offering advantages over established therapies. This review utilizes synthesized data from peer-reviewed clinical trials, systematic reviews, meta-analyses, regulatory documents, and international guidelines. Only studies involving adult patients with RA treated by approved JAKis (baricitinib, tofacitinib, upadacitinib, peficitinib, and filgotinib) were included. Both real-world observational studies and randomized controlled trials were assessed for efficacy, safety, drug interaction, and long-term outcomes. JAKis consistently demonstrated superior responses compared with placebo and methotrexate, with higher American College of Rheumatology 20% response rates, improved disease activity scores, reduced radiographic progression, and enhanced patient-reported outcomes. In head-to-head comparisons, baricitinib and upadacitinib demonstrated advantages over adalimumab across multiple efficacy domains. However, safety concerns emerged, particularly regarding major adverse cardiovascular events, herpes zoster, and malignancy. While randomized controlled trials showed low absolute event rates, observational studies revealed higher risks compared with tumor necrosis factor inhibitors, especially among older patients, smokers, and those with cardiovascular comorbidities. JAKis represent a highly effective and convenient therapeutic option in RA management, offering significant improvements over csDMARDs and certain biologic agents. Nonetheless, their use requires individualized, risk-stratified decision-making, with particular caution in patients at elevated cardiovascular or malignancy risk. Ongoing long-term studies and real-world data remain essential to further define their benefit-risk profile and optimize their integration into personalized RA care.

## Introduction and background

Rheumatoid arthritis (RA) is an autoimmune, chronic, systemic disease characterized by autoantibody production, progressive joint erosion, and persistent synovitis that leads to pain, swelling, disability, escalating morbidity, and mortality [1,2].

The Global Burden of Disease 2021 study estimated that RA affected 17.6 million people worldwide in 2020, with an adult age‑standardized prevalence of approximately 208.8 cases per 100,000 population, representing a 14% increase since 1990. The total disease burden remains substantial: in 2020, there were about 3.06 million disability‑adjusted life‑years attributable to RA, the majority of which (over 75%) were due to years lived with disability. Without improvements in early diagnosis and access to treatment, the number of people living with RA is forecast to rise to over 31 million by 2050, underscoring its increasing global health impact [3].

**Figure 1:**
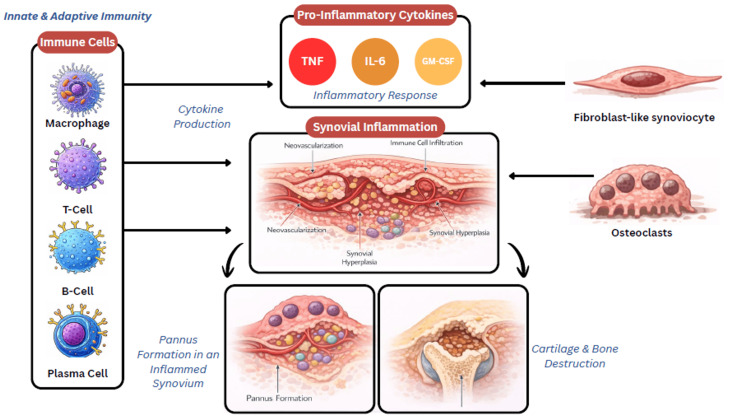
Pathogenesis of RA RA: rheumatoid arthritis, TNF: tumor necrosis factor, IL-6: interleukin-6, GM-CSF: granulocyte-macrophage colony-stimulating factor Image Credit: Author. Created using Adobe Photoshop (Adobe Inc., San Jose, CA, USA)

The pathogenesis of RA involves multiple interactions among innate and adaptive immune cells, with fibroblast-like synoviocytes, macrophages, T and B lymphocytes, and osteoclasts driving a cytokine-rich inflammatory reaction dominated by interleukin-6 (IL-6), granulocyte-macrophage colony-stimulating factor (GM-CSF), and tumor necrosis factor (TNF), which ultimately promotes pannus formation and joint erosion (Figure 1) [1,4,5].

Standard treatment follows a treat-to-target approach that aims to lower disease activity or achieve remission, starting with conventional synthetic disease-modifying antirheumatic drugs (csDMARDs) such as sulfasalazine, methotrexate (MTX), leflunomide, and hydroxychloroquine, often combined with short-term glucocorticoids [6]. If the disease response is unsatisfactory despite the use of csDMARDs, guidelines recommend escalating to biologic DMARDs (bDMARDs) such as IL-6 receptor inhibitors, TNF inhibitors (TNFis), CTLA-4 Ig, and anti-CD20 monoclonal antibodies, or to targeted synthetic DMARDs (tsDMARDs), including JAK inhibitors (JAKis), which are increasingly used in the management of RA [6].

Using JAKis to treat RA offers a different mechanism of action than that of bDMARDs. They act by modulating the immune-inflammatory response by blocking JAK enzymes, thereby reducing RA-associated symptoms and signs, such as swelling, pain, stiffness, and joint destruction [7,8].

Janus kinase inhibitors

JAKis are small-molecule therapeutics administered orally and reduce inflammation associated with inflammatory and autoimmune diseases by moderating the JAK-signal transducer and activator of transcription (STAT) pathway [7].

Janus kinases are essential intracellular tyrosine kinases that mediate immune responses by transmitting signals from a wide range of cytokine receptors that lack intrinsic kinase activity. When cytokines such as interleukins or interferons bind to their receptors, the associated JAKs become activated and phosphorylate both the receptor and STAT proteins. Activated STATs then dimerize, translocate to the nucleus, and hence regulate gene transcription that controls immune cell development, survival, proliferation, and function. Through this JAK-STAT pathway, JAKs play a central role in both innate and adaptive immunity; hence, defects in JAK signaling lead to impaired immune responses and immunodeficiency, underscoring their critical importance in normal immune regulation. Accordingly, abnormal JAK activity contributes to immune diseases primarily by disrupting normal cytokine signaling, leading either to immune deficiency or immune overactivation [8].

Moreover, they act by blocking JAK family enzymes (TYK2, JAK1, JAK2, JAK3), thereby preventing STAT phosphorylation and downstream transcription of pro-inflammatory cytokine-responsive genes [8,9]. By inhibiting multiple cytokine pathways simultaneously (including IL-6, interferons, and GM-CSF), JAKis provide a broader immunomodulatory effect than single-cytokine blockade [9].

The first JAKi approved by the FDA for use in patients with RA was tofacitinib (brand name: Xeljanz) in 2012, followed by baricitinib (brand name: Olumiant) in 2018 and upadacitinib (brand name: Rinvoq) in 2019 in the United States [10-12]. In Europe, filgotinib was approved in 2020, whereas peficitinib was authorized in Japan in 2019 [11,12]. Current international guidelines classify JAKis as tsDMARDs and recommend their use within a safety-conscious, shared decision-making framework [5,6].

This review aims to provide an updated synthesis of the evidence on the role of JAKis in the management of RA. While csDMARDs and bDMARDs remain the cornerstone of RA treatment, limitations such as incomplete efficacy, loss of response over time, parenteral administration, and adverse events necessitate alternative therapeutic approaches [1,5,6]. JAKis, as targeted synthetic DMARDs, are available for oral administration, have a rapid onset of action, and broadly modulate the cytokine pathway, thereby distinguishing them from biologic therapies that target individual cytokines [8,9]. Furthermore, this review aims to critically examine the mechanisms of action, clinical efficacy, safety profiles, and regulatory considerations of currently approved JAKis in RA [10-14]. Particular attention is given to comparative effectiveness relative to established biologic therapies and to emerging safety concerns [5,6]. Additionally, it will highlight the evolving role of JAKinhibitors in international treatment guidelines and their implications for personalized medicine in RA management [5,6].

Methods

**Figure 2:**
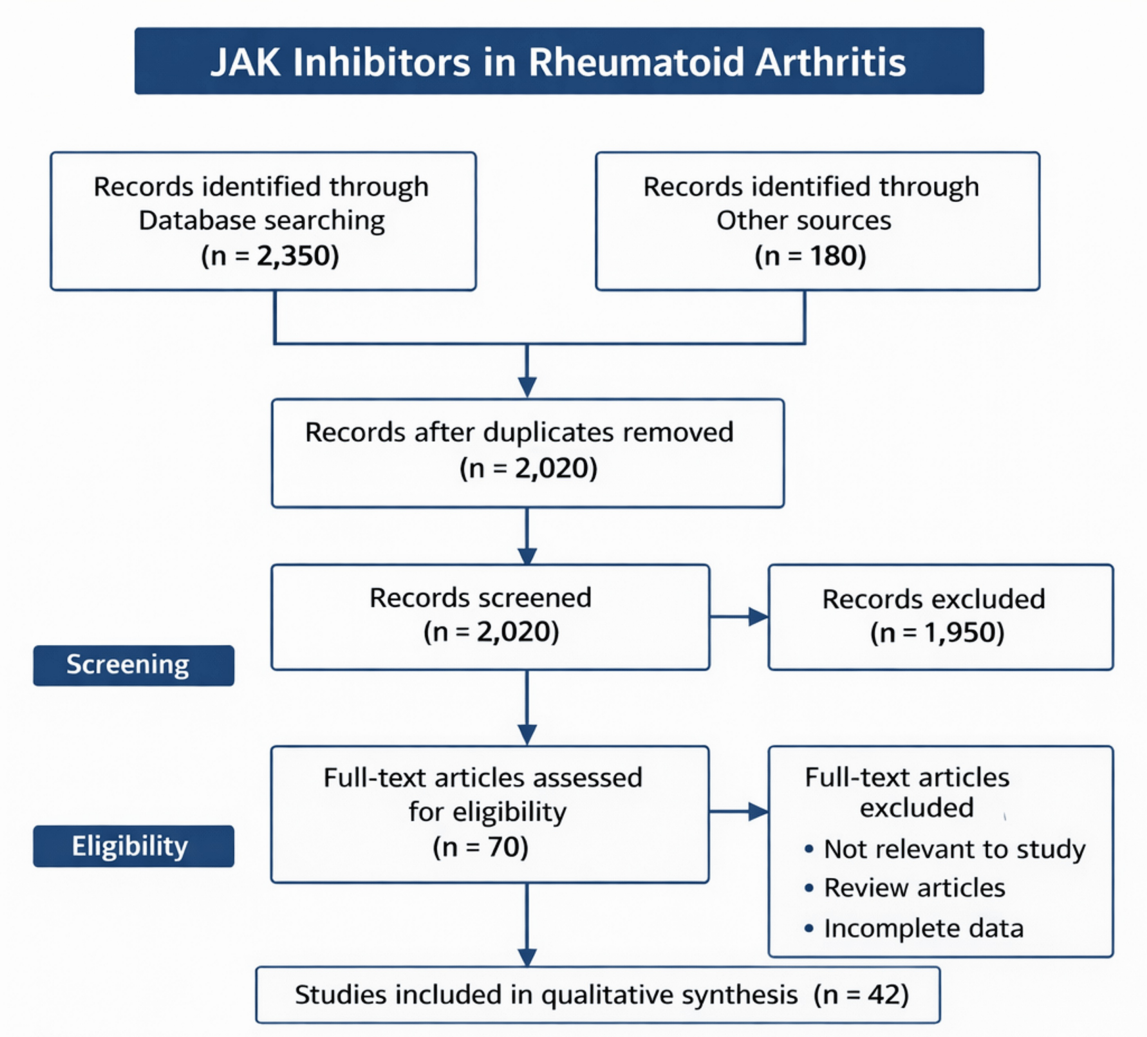
PRISMA flowchart PRISMA: Preferred Reporting Items for Systematic reviews and Meta-Analyses

This review was limited to peer-reviewed publications, clinical trials, regulatory documents, and international treatment guidelines addressing the role of JAKis in RA management. Only studies involving adult patients (≥18 years) with a confirmed diagnosis of RA based on ACR/EULAR criteria were considered. Both observational studies and randomized controlled trials were included to capture efficacy, safety, and real-world outcomes. Systematic reviews and meta-analyses were also included where relevant to provide higher levels of evidence (Figure 2).

The review boundaries were restricted to JAKis that have received regulatory approval for RA treatment, specifically baricitinib, tofacitinib, peficitinib, upadacitinib, and filgotinib. Studies involving other investigational JAKis not yet approved for RA, or those approved solely for other immune-mediated conditions, were excluded.

Exclusion criteria included non-English language publications, conference abstracts without full-text availability, preclinical animal or in vitro studies, and reports focused exclusively on diseases other than RA (e.g., psoriatic arthritis, ulcerative colitis, atopic dermatitis). Commentary, editorials, and opinion papers without primary or secondary data synthesis were also excluded.

## Review

Superiority of JAKis over other DMARDs

JAKis vs Placebo in RA

A significant advancement in the treatment of RA has been the introduction of JAKis. Several randomized controlled trials (RCTs) have compared JAKis with placebo, with the American College of Rheumatology 20% response rate (ACR20) often serving as the primary benchmark of efficacy. Other clinical and patient-reported outcomes (PROs), including the Health Assessment Questionnaire Disability Index (HAQ-DI), Disease Activity Score in 28 joints (DAS28), Patient’s Global Assessment (PtGA), the 36-Item Short Form Survey (SF-36), Functional Assessment of Chronic Illness Therapy Fatigue (FACIT-F), and the EQ-5D index, have also been utilized to establish their therapeutic potential.

The first JAKi approved for the treatment of RA was tofacitinib. A pivotal trial evaluated tofacitinib monotherapy in adults with active RA, where subjects were randomly assigned to receive tofacitinib 5 mg or 10 mg twice daily, or placebo. At three months, both tofacitinib doses achieved substantially higher ACR20 (60-66%) compared with placebo (27%). Moreover, the physical function, as measured by HAQ-DI, improved significantly in the tofacitinib groups. However, rates of DAS28-ESR remission (<2.6) at three months did not differ significantly from placebo [15]. In another study investigating MTX combined with tofacitinib in patients with an inadequate response to MTX alone, at six months, ACR20 was higher with tofacitinib 5 mg (52%) and 10 mg (53%) than with placebo (28%). Moreover, HAQ-DI and DAS28-ESR remission rates were higher with active treatment [16].

Baricitinib was the second JAKi to gain FDA approval for the management of RA. In a phase 3 trial in patients with active RA receiving background MTX, baricitinib achieved an ACR20 response in 70% of patients, compared with 40% in the placebo group (p < 0.001). Baricitinib also produced significant improvements in HAQ-DI, DAS28, Simplified Disease Activity Index remission rates, and radiographic progression by week 24, with a mean change in modified Total Sharp Score (mTSS) of 0.41 vs 0.90 for placebo (p < 0.001) [17]. Further assessment of PROs was conducted in this cohort, in which baricitinib significantly improved pain, PtGA, fatigue, physical function, and health-related quality-of-life measures, including EQ-5D and SF-36. These benefits were sustained up to 52 weeks of treatment [18].

The third JAKi, upadacitinib, was approved in 2019. A 2024 meta-analysis and systematic review assessed the efficacy of all JAKis vs placebo and csDMARDs and reported numerous positive results. At 12 weeks, JAKis consistently outperformed placebo, with ACR20 relative risks (RRs) ranging from 1.74 to 3.08. The Surface Under the Cumulative Ranking Curve (SUCRA) analysis identified upadacitinib at both 15 mg and 30 mg as particularly effective. At 24 weeks, JAKis remained superior to placebo or csDMARDs, with ACR20 RRs ranging from 1.16 to 1.86. Among the highest-ranking regimens were baricitinib 4 mg with csDMARD and upadacitinib 15 mg with csDMARD [19].

Another meta-analysis comparing the efficacy of the approved JAKis in the management of RA showed that in patients with RA who responded inadequately to MTX, two lines of management, including baricitinib 4 mg combined with MTX and upadacitinib 15 mg combined with MTX, have the highest ACR response rates [20].

Collectively, these studies demonstrate that JAKis are superior to placebo in patients with RA, improving both PROs and clinical disease activity. Although differences exist in remission rates and safety profiles, consistent findings across multiple trials affirm their efficacy in RA management.

*JAKis *vs* MTX in RA*

**Figure 3:**
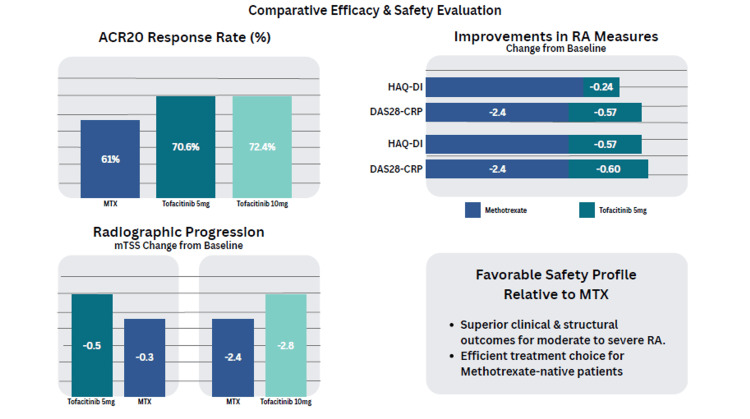
JAKis vs MTX in RA JAKis: janus kinase inhibitors, RA: rheumatoid arthritis, MTX: methotrexate, ACR20: American College of Rheumatology 20% response rate, mTSS: modified Total Sharp Score, HAQ-DI: Health Assessment Questionnaire Disability Index, DAS28-CRP: Disease Activity Score in 28 joints using C-reactive protein

JAKis have also been evaluated in comparison to MTX, which is the most utilized drug in the treatment of RA [21]. In clinical studies, tofacitinib monotherapy was found to be better than MTX in improving the symptoms and signs of RA, as well as preventing the progression of structural joint damage [21]. The ACR20 was 70.6% for tofacitinib 5 mg BID, 72.4% for tofacitinib 10 mg BID, and 61.0% for MTX. Improvements were also observed in functional and disease activity measures: HAQ-DI scores decreased by -0.57 and -0.60 for tofacitinib 5 mg and 10 mg BID, respectively, compared with -0.44 for MTX; DAS28-CRP scores decreased by -2.7 and -2.8 for the two tofacitinib doses, compared with -2.4 for MTX. Radiographic progression, assessed by mTSS, showed minimal worsening with tofacitinib (-0.3 and -0.2) compared with MTX (-0.5) [22]. These results demonstrate that both doses of tofacitinib monotherapy are superior to MTX in improving clinical and structural outcomes in MTX-naive patients with moderate to severe RA. Additionally, tofacitinib exhibited a favorable safety profile compared with MTX, supporting its role as an efficient treatment option for patients who have not previously received MTX (Figure 3) [22].

*JAKis *vs* Adalimumab in RA*

**Figure 4:**
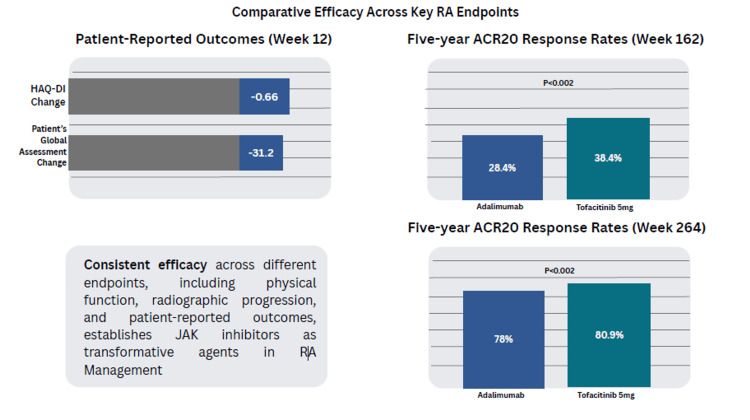
JAKis vs adalimumab in RA JAKis: janus kinase inhibitors, RA: rheumatoid arthritis, ACR20: American College of Rheumatology 20% response rate, HAQ-DI: Health Assessment Questionnaire Disability Index

Baricitinib demonstrated statistically significant improvements in most prespecified PROs compared with adalimumab at week 12 [23]. HAQ-DI scores for baricitinib improved by −0.66 vs −0.56 for adalimumab, while PtGA scores improved by −31.2 vs −26.6, respectively (Figure 4) [23].

In a long-term study comparing upadacitinib and adalimumab over five years, efficacy was assessed using radiographic outcomes and clinical response. The percentages were 80.9% for patients on continuous upadacitinib and 78.0% for patients on continuous adalimumab [24]. In mTSS, the mean change from baseline at week 192 was 0.53 for upadacitinib and 1.18 for adalimumab. At week 264, the ACR20 was 38.4% with upadacitinib vs 28.4% with adalimumab (p = 0.002) (Table 1) [24].

**Table 1:** Summary of efficacy outcomes between JAKis and other DMARDs in RA JAKis: janus kinase inhibitors, BID: twice daily, ACR20: American College of Rheumatology 20% response rate, HAQ-DI: Health Assessment Questionnaire Disability Index, MTX: methotrexate, DAS28-ESR: Disease Activity Score in 28 joints using erythrocyte sedimentation rate, DAS28: CRP: Disease Activity Score in 28 joints using C-reactive protein, ESR: erythrocyte sedimentation rate, CRP: C-reactive protein, mTSS: Modified Total Sharp Score, RR: risk ratio, csDMARDs: conventional synthetic disease-modifying antirheumatic drugs, SUCRA: surface under the cumulative ranking curve, PtGA: Patient's Global Assessment

Comparator	JAKi (dose/regimen)	Timepoint	Key efficacy outcomes	Comparator outcomes	Statistical superiority
Placebo	Tofacitinib 5-10 mg BID (monotherapy)	3 months	ACR20: 60-66%, HAQ-DI: significant improvement	ACR20: 27%	Yes (ACR20, HAQ-DI)
	Tofacitinib + MTX	6 months	ACR20: 52-53%, DAS28-ESR remission: improved	ACR20: 28%	Yes
	Baricitinib + MTX	24 weeks	ACR20: 70%, mTSS: 0.41	ACR20: 40%, mTSS: 0.90	Yes (p < 0.001)
	All JAKis (meta-analysis)	12 weeks	ACR20 RR: 1.74-3.08	Placebo	Yes
		24 weeks	ACR20 RR: 1.16-1.86	Placebo/csDMARDs	Yes
MTX	Tofacitinib 5 mg BID	24 months	ACR20: 70.6%, HAQ-DI: −0.57, DAS28-CRP: −2.7, mTSS: −0.3	ACR20: 61.0%, HAQ-DI: −0.44, DAS28-CRP: −2.4, mTSS: −0.5	Yes
	Tofacitinib 10 mg BID	24 months	ACR20: 72.4%, HAQ-DI: −0.60, DAS28-CRP: −2.8, mTSS: −0.2	Same as above	Yes
csDMARDs	Upadacitinib or baricitinib + csDMARD (meta-analysis)	24 weeks	Highest-ranked regimens by SUCRA	csDMARDs alone	Yes
Adalimumab	Baricitinib	12 weeks	HAQ-DI: −0.66, PtGA: −31.2	HAQ-DI: −0.56, PtGA: −26.6	Yes
	Upadacitinib	192 weeks	No radiographic progression: 80.9%, mTSS: 0.53	78.0% mTSS: 1.18	Yes
		264 weeks	ACR20: 38.4%	28.4%	Yes (p = 0.002)

The accumulated evidence from long-term studies and randomized controlled trials clearly establishes the superiority of JAKis over placebo, MTX, and even bDMARDs such as adalimumab in RA management. Across multiple efficacy endpoints, including ACR20, disease activity scores, physical function, radiographic progression, and PROs, JAKis consistently demonstrate faster and more robust improvements. Importantly, they yield meaningful improvements in both clinical outcomes and quality of life, underscoring their role as transformative agents in the treatment of RA. While safety considerations remain essential, the consistent efficacy across different patient populations confirms JAKis as a highly effective therapeutic option, offering an advantage over csDMARDs and bDMARDs in achieving disease control and improving long-term outcomes.

Adverse effects of JAKis

Some risk factors for developing serious complications should be considered when prescribing oral JAKis for RA patients. Some studies show potential risks of JAKis, such as major cardiovascular problems like heart attacks or strokes, deep vein thrombosis, pulmonary embolism, cancer, serious infections, upper respiratory infections, nasopharyngitis, headache, nausea, and acne [25]. Significant weight gain may also occur in patients taking JAKi due to reduced muscle wasting, as the JAK-STAT pathway is inhibited [26]. Moreover, some neurological adverse effects may be experienced by RA patients managed with JAKi due to JAK-STAT pathway inhibition, such as dizziness, dysarthria, aphasia, ataxia, peripheral neuropathy, amnesia, and Wernicke encephalopathy [27]. Additionally, JAKis’ side effects can occur at any time, as their risk of side effects is not dose-dependent, such as in steroid intake, for example [28]. In trials evaluating long-term side effects, results varied widely depending on the JAKi used to treat RA. However, studies have shown a strong association between JAKis and venous thromboembolism (VTE), particularly when used for prolonged periods, such as one year or more [28].

Overall, JAKis may adversely affect the immune system and cause common or rare side effects. The most common JAKi’s side effects comprise nausea, headache, and respiratory infections [29]. However, serious JAKis’ side effects such as stroke, myocardial infarction, pulmonary embolism, deep vein thrombosis, and risk of malignancy are rare but possible [29]. Hence, despite the inconsistent data, a subpopulation at high risk of JAKi adverse effects is indicated by some cumulative studies, including patients with cardiovascular diseases, stroke, the elderly over 65 years of age, smokers, and patients with malignancy risk factors [29]. Hence, a more detailed overview is provided below for these high-risk groups that might be adversely affected by JAKi, including the associated adverse effects.

Major Adverse Cardiovascular Effects

Specific concerns have been raised regarding JAKis’ long-term safety, particularly with respect to major adverse cardiovascular effects (MACE), which encompass stroke, myocardial infarction, and cardiovascular death [30]. Most studies have highlighted the safety profiles of specific JAKis compared with TNFis.

In a high-cardiovascular-risk RA population, tofacitinib was associated with a higher rate of MACE than TNFi. One study found a MACE incidence of 3.4% with tofacitinib vs 2.5% with a TNFi over a median of 4.0 y, with a hazard ratio of 1.33 (95% CI 0.91-1.94) [30]. Another cohort study reported MACE incidence rates (IR/100 patient-years) of 2.56 with JAKi vs 0.83 with TNFi [31].

Outside of high-risk settings, RCT/meta-analytic data across trials (various immune diseases) showed no statistically significant difference in MACE for JAKis vs placebo overall; JAKis vs adalimumab showed higher all-cause mortality, but the MACE difference was not significant (MACE are rare in RCTs with shorter follow-up) [25]. However, several real-world cohorts report higher cardiovascular/MACE rates vs TNFi, suggesting that if a difference exists, it is most apparent against TNFi comparators and in patients with cardiovascular risk [31].

Taken together, the current evidence suggests that the risk of MACE with JAKis is context-dependent. The strongest safety signal arises from high-risk cardiovascular populations, in which tofacitinib demonstrated a numerically higher incidence of MACE than TNFis. Observational data reinforce this trend, consistently reporting higher MACE rates with JAKis than with TNFi, particularly in older patients and those with established cardiovascular comorbidities. Conversely, randomized controlled trials in broader or lower-risk populations have generally shown low absolute event rates and no statistically significant excess risk compared with placebo. This indicates that while JAKis remain a valuable therapeutic option, careful patient selection and cardiovascular risk stratification are critical to optimize safety in clinical practice.

Herpes Zoster

Another major concern was regarding the risk of herpes zoster (HZ) infection. Meta-analyses and pooled analyses show an increased HZ risk with JAKis compared with placebo, MTX, or TNFi; typically, the risk is approximately twofold to fourfold higher, depending on the drug, dose, and comparator [32]. Reviews and systematic analyses consistently identify HZ as a reproducible safety signal for the class [33].

When comparing different JAKis, tofacitinib had an incidence rate of 3.6 per 100 patient-years, which is several-fold higher than typical RA background rates [34]. Baricitinib, on the other hand, had an incidence rate of 3.1-4.4 per 100 per year, and the incidence rate increased as the dose increased (2 mg: ≈3.1/100 PY; 4 mg: ≈4.4/100 PY) [35]. Upadacitinib trial data showed a dose-dependent relationship: the incidence rate for 15 mg was 3.0/100 PY, whereas for 30 mg it was 5.3/100 PY in RA trials. Lower dose regimens (15 mg) still showed higher rates than placebo/MTX [36].

Overall, HZ represents a consistent and clinically relevant adverse effect of JAKi therapy. Across trials and meta-analyses, the class is associated with a two- to fourfold higher risk of HZ infection compared with placebo, MTX, or TNFis. Hence, although incidence rates vary across agents and doses, they consistently exceed background RA rates, with higher exposures generally associated with greater risk. Notably, while most adverse effects are nonserious and localized, the reproducibility of this safety signal across different JAKis underscores the need for careful patient selection, dose optimization, and consideration of HZ vaccination prior to initiating therapy.

Malignancy

JAKis are associated with a small but reproducible increase in malignancy risk when compared with TNFi, though they are not clearly increased vs placebo or MTX in randomized trial data.

A trial studying malignancy risk (with the exclusion of non-melanoma skin cancer (NMSC)) demonstrated an incidence of 4.2% in patients on combined tofacitinib doses vs 2.9% on TNFi. Hazard ratio (tofacitinib vs TNFi) for malignancies excluding NMSC after month 18: HR 1.93 (95% CI 1.22-3.06) (risk curves diverged after month 18). The early period (baseline to month 18) showed no difference (HR 0.93 (0.53-1.62)). The trial, therefore, identified a long-term excess that emerged over time. The most common malignancy subtype associated with tofacitinib was lung cancer, which was proportionately increased with the higher (10 mg BID) dose and in smokers as well [37].

A meta-analysis and systematic review reported an overall incidence of malignancy of 1.15 per 100 person-years in RCTs [38]. Real-world registries and claims studies report mixed results: some show no difference relative to TNFi after adjustment, whereas others show small increases for specific cancer types (e.g., NMSC or lung cancer in certain subgroups). Taken together, these data add uncertainty about generalizability beyond the ORAL Surveillance population [38].

The absolute risk of new malignancy events on JAKis in trials was low (1-1.3 events per 100 patient-years overall). Still, the relative increase compared with TNFi was consistent across pooled analyses (IRR = 1.5). In ORAL Surveillance, the absolute difference was 1.3% (4.2% vs 2.9%) over four years [37].

The signal was concentrated in a higher-risk population (older patients with cardiovascular risk factors), emerged with longer follow-up (>18 months), and appeared stronger for specific cancer subtypes (notably lung cancer) and in smokers, suggesting effect modification by baseline risk and exposures [37].

For average-risk patients (younger, no major cardiovascular or cancer risk factors), randomized trial data did not show a clear increase in malignancy risk with JAKis vs placebo or MTX [38].

For older patients with cardiovascular risk factors, current or prior smokers, or other cancer risk factors, clinicians should weigh the small absolute increase in malignancy tendency observed with tofacitinib (and the class signal vs TNFi) when choosing therapy and consider alternative agents (e.g., TNFi) where appropriate [38].

Venous Thromboembolism

A systematic review and meta-analysis published in JAMA Network Open evaluated the comparative safety of JAKis vs TNF antagonists in adults with immune-mediated inflammatory diseases. Across real-world and observational studies, the authors found no significant differences between JAKis and TNF antagonists in the risk of serious infections, malignancy (excluding NMSC), or major adverse cardiovascular events [39]. However, JAKis were associated with a modestly increased risk of VTE compared with TNF antagonists, although absolute event rates were low [39]. Overall, the findings suggest that the safety profiles of JAKis and TNF antagonists are broadly comparable, with the main safety signal being a small increase in thromboembolic risk with JAKis, hence supporting their use with careful patient selection and risk assessment [39].

The safety profile of JAKis reveals important class-specific risks that must be carefully considered in clinical decision-making. While major adverse cardiovascular events appear most pronounced in older patients with pre-existing cardiovascular risk factors, particularly when compared with TNFis, randomized trials in broader populations show low absolute event rates. HZ, however, represents a consistent and reproducible adverse effect across all JAKis, with risk magnitude influenced by dose and agent, hence underscoring the importance of vaccination strategies and risk mitigation before treatment initiation. Malignancy risk, though generally low in absolute terms, emerges with longer-term exposure and is most evident in higher-risk groups such as smokers and older patients with cardiovascular comorbidities; hence, again showing a stronger signal compared with TNFi. Collectively, these findings highlight that JAKis remain effective therapeutic options in RA but require individualized risk-benefit assessment, careful patient selection, and proactive monitoring to optimize both efficacy and safety.

JAKi drug interactions

Some studies have shown that JAKis, especially Rinvoq, interact with certain antibiotics (such as rifampin and clarithromycin), certain antifungals, diltiazem and verapamil, medications containing ritonavir, some herbal medicines (such as St. John’s Wort), and some fruits (such as grapefruit). Hence, when prescribing JAKis to RA patients, they should be warned about the above-mentioned interactions to avoid complications or even reduce the efficacy of the drugs [40].

Researchers have reported that numerous fruits, such as pomegranate, blackberry, strawberry, rosehip, boysenberry, and feijoa, are potent JAK2 inhibitors because they contain ellagitannins, known kinase inhibitors [41]. Some researchers have also reported curcumin’s anti-inflammatory effect by inhibiting signaling through the JAK-STAT pathway; hence, curcumin’s protective effects are employed [42].

## Conclusions

JAKis have emerged as a transformative therapeutic class in the management of RA, offering rapid onset of action, oral administration, and broad modulation of the cytokine pathway. Robust clinical evidence demonstrates their superiority over placebo, MTX, and established biologic agents, such as adalimumab, in improving disease activity, physical function, and radiographic outcomes. These attributes establish JAKis as effective alternatives for patients who have an inadequate response to or intolerance of csDMARDs.

Nevertheless, their expanding use is tempered by safety concerns, including an increased incidence of major adverse cardiovascular events, HZ infection, and malignancy, particularly among older patients, smokers, and individuals with pre-existing risk factors. Although absolute event rates remain relatively low in randomized trials, emerging long-term observational data indicate higher risks than those observed with TNFis, underscoring the importance of meticulous patient selection and ongoing monitoring. Continued real-world studies and extended follow-up are essential to refine the long-term benefit-risk profile and inform evidence-based treatment algorithms.
